# Transmembrane Protein 97 (TMEM97): Molecular Target and Treatment in Age-Related Macular Degeneration (AMD)

**DOI:** 10.3390/biom15091228

**Published:** 2025-08-26

**Authors:** Alyssa Stathopoulos, Joshua J. Wang, Stephen F. Martin, Sarah X. Zhang

**Affiliations:** 1 Department of Ophthalmology, Ira G. and Ross Eye Institute, Jacobs School of Medicine and Biomedical Sciences, University at Buffalo, The State University of New York, 955 Main Street, Buffalo, NY 14203, USA; arstatho@buffalo.edu (A.S.); xzhang38@buffalo.edu (S.X.Z.); 2 Department of Chemistry, The University of Texas at Austin, Austin, TX 78712, USA; sfmartin@mail.utexas.edu

**Keywords:** retinal degeneration, small molecule, molecular target and treatment, transmembrane protein 97

## Abstract

Age-related macular degeneration (AMD) is a common eye disease that significantly affects daily activities and impedes the quality of life in aging adults, yet effective treatments to halt or reverse disease progression are currently lacking. Ongoing research aims at understanding the complex mechanisms underlying AMD pathophysiology involving retinal pigment epithelium (RPE) dysfunction, drusen formation, inflammation, neovascularization, and RPE/photoreceptor degeneration. Sigma 2 receptor/transmembrane protein 97 (σ_2_R/TMEM97) is a multifunctional protein implicated in cellular processes including cholesterol homeostasis, lysosome-dependent autophagy, calcium homeostasis, and integrated stress response (ISR). Recent genome-wide association studies (GWASs) have identified σ_2_R/TMEM97 as a novel genetic risk factor strongly associated with AMD development. In this review, we summarize recent research progress on σ_2_R/TMEM97 in age-related neurodegenerative diseases, highlighting its implication as a molecular target in AMD via regulating oxidative stress, inflammation, lipid uptake, drusen formation, and epithelial–mesenchymal transition (EMT). We also discuss the potential of modulating σ_2_R/TMEM97 function with novel small-molecule drugs as a promising treatment for dry AMD and the unresolved questions in understanding the mechanistic basis of their actions.

## 1. Introduction

Age-related macular degeneration (AMD) is a leading cause of vision loss in aging adults, and its global prevalence has increased substantially in recent years [1]. In 2020, AMD accounted for 6–9% of all cases of legal blindness, affecting 196 million people worldwide; this number is projected to increase to 288 million by 2040 [2]. AMD typically affects patients aged 50 years or older, characterized by progressive degeneration of the macula, a small area of the retina responsible for central vision [3,4]. Hallmark pathological changes of AMD include the formation of drusen, manifested as yellow deposits under the retina, and RPE abnormalities (e.g., pigment changes) in early AMD, focal RPE and photoreceptor degeneration, and/or macular neovascularization (MNV) in the late stage of the disease; the latter is classified into (1) geographic atrophy (GA) or dry AMD and (2) neovascular AMD or wet AMD based on the absence or presence of MNV [3,4]. Other atypical manifestations include polypoidal choroidal vasculopathy (PCV), reticular pseudodrusen, or retinal angiomatous proliferation [3,4]. Current standard treatment for wet AMD primarily relies on anti-VEGF medications [3,4,5], with two new drugs, Syfovre (pegcetacoplan) and Izervay (avacincaptad pegol), being approved by the FDA in 2023 for the treatment of GA [6,7]. Notably, all these therapies target advanced forms of AMD and are delivered via intraocular injection, which is an invasive procedure that could cause severe complications such as endophthalmitis [8]. Thus, developing small-molecule drugs and non-invasive therapies for patients with early or intermediate AMD to prevent, halt, or reverse disease progression and vision loss is an unmet need.

The lack of effective treatments for AMD is in part due to the complex nature of the disease that involves an interplay of multiple etiological factors and signaling pathways, including genetic susceptibility, environmental risk factors, oxidative stress, mitochondrial dysfunction, impaired lipid metabolism, chronic inflammation, and immune system dysregulation [9,10,11]. The normal aging process and cumulative age-related changes, such as declining function of the integrated regulatory systems that maintain protein and lipid homeostasis, metabolic function, and redox status, can cause RPE dysfunction and promote lipid-rich drusen deposits in early AMD [3,12,13,14]. Recent large-scale genetic and genomic studies have identified genetic variants associated with AMD pathophysiology [15,16], among which is sigma 2 receptor/transmembrane protein 97 (σ_2_R/TMEM97), also known as meningioma-associated protein (MAC30) [17,18]. σ_2_R/TMEM97 is a transmembrane protein of the endoplasmic reticulum (ER) [19] involved in cellular processes including cholesterol homeostasis, lipid metabolism, lysosome autophagy, calcium homeostasis, and integrated stress response (ISR) [20,21,22,23]. σ_2_R/TMEM97 is highly expressed in neurons and cancer cells [24], and small-molecule allosteric modulators of σ_2_R/TMEM97 have demonstrated robust neuroprotective effects in various neurological conditions and diseases [25,26,27,28,29]. Recent work has also identified a role of σ_2_R/TMEM97 in AMD pathogenesis through the regulation of oxidative stress, inflammation, and epithelial–mesenchymal transition (EMT) of RPE cells [30,31,32]. In this review, we will discuss recent progress on σ_2_R/TMEM97 research in neurodegeneration, focusing on its implication in AMD-related signaling pathways and highlighting the therapeutic potential of σ_2_R/TMEM97 modulators in the treatment of AMD.

## 2. AMD: Clinical Manifestations, Treatment, and Pathophysiology

AMD is a chronic disease classified into three stages: early, intermediate, and advanced AMD. The progression of AMD is a slow process that takes years to reach the later or advanced stage [3,4]. Early AMD is characterized by small drusen, a small amount of intermediate drusen, or mild RPE abnormalities [4]. Patients with early AMD often have no symptoms of vision impairment. Intermediate AMD is characterized by a large amount of intermediate drusen, a small amount of large drusen, or an atrophic area, which can affect the central vision [4]. Advanced AMD is characterized by progressive loss of the RPE and photoreceptors in the macula, possibly involving the fovea, neovascular maculopathy, or disciform scar, resulting in irreversible central vision loss [3,4]. According to the presence or absence of aberrant new vessel growth in the macula, AMD is divided into two major types: dry AMD and wet AMD. Dry AMD is characterized by drusen and focal RPE/photoreceptor atrophy and accounts for approximately 85% to 90% of AMD cases [33]. While both types of AMD severely impact patients’ activities of daily living and quality of life, dry AMD is more detrimental due to the progressive degeneration and central vision loss, as well as the lack of available treatments [33].

Despite recent advances, the management and treatment options for early and intermediate AMD are limited, including observation, risk factor reduction, and dietary supplementation with minerals and vitamins [3,4,33]. According to the Age-Related Eye Disease Study (AREDS), dietary supplementation with antioxidants and zinc can decrease the rates of progression of intermediate AMD to advanced AMD [34]. The AREDS2 study shows that replacing beta-carotene, which was found to increase the risk of lung cancer in smokers, with lutein and zeaxanthin in the AREDS formulation can also reduce the risk of advanced AMD, but omega-3 does not offer additional protection [35,36]. Nevertheless, these supplements provide options to AMD patients with different systemic conditions (e.g., smokers and non-smokers) to select the most effective regimen for slowing down AMD progression. In 2023, the FDA approved two intravitreal injections to treat GA. On 17 February 2023, the FDA approved Syfovre (Apellis), a pegcetacoplan injection that acts as a complement C3 inhibitor, to be used for the treatment of GA [6,7]. On 4 August 2023, the FDA approved Izervay (Astellass), an avacincaptad pegol injection that acts as a complement C5 inhibitor, to treat GA [37,38]. These two drugs are the first FDA-approved treatments for advanced dry AMD. Treatment options for wet AMD include anti-VEGF agents, photodynamic therapy (PDT), and laser photocoagulation [3,4]. First-line treatment for wet AMD is anti-VEGF therapies (aflibercept, bevacizumab, brolucizumab, and ranibizumab) that are typically administered via intravitreal injection to maximize the efficacy and preserve visual acuity [3,4,5]. New treatments, including gene therapy, stem cell therapy, and complement system targets, are being investigated [3,4]. Because all current treatments target advanced AMD and the drugs are delivered by invasive intravitreal injections, there is an unmet need for developing novel non-invasive small-molecule therapies that can be taken orally or administered topically to treat AMD, particularly dry AMD.

Drusen formation is a major characteristic of early AMD. The landmark Beaver Dam Eye Study (BDES) examined the fifteen-year cumulative incidence of AMD fundus changes in nearly 4000 individuals from 1988 to 2005 [39]. The study found that eyes with intermediate (or soft) indistinct drusen or pigmentary abnormalities at baseline were more likely to develop late or advanced AMD [39]. Drusen are extracellular deposits primarily found between the RPE and Bruch’s membrane (BrM) [3], containing a variety of lipids, specifically large, apolipoprotein B (ApoB)- and apolipoprotein E (ApoE)-containing, cholesterol-rich lipoproteins secreted by the RPE [40,41]. Normally, the RPE acquires cholesterol from lipoproteins in the circulation or via phagocytosis of photoreceptor outer segments (POSs), recycles cholesterol back to photoreceptors, or eliminates it via a high-density lipoprotein particle [41]. When this reverse cholesterol transport is impaired, the RPE continues to accumulate cholesterol and secrete ApoB100 lipoproteins into the Bruch’s membrane. The secreted lipids build up between the RPE basal lamina space and Bruch’s membrane due to impaired transport across an aged Bruch’s membrane [41]. This process, along with the accumulation of cellular debris as waste deposits, contributes to drusen formation between the RPE and Bruch’s membrane [41].

AMD is a multifactorial disease involving genetic and environmental risk factors such as aging, smoking, previous cataract surgery, and systemic diseases [3,4,42]. Recent genome-wide association studies (GWASs) identified numerous genetic variants, including TMEM97, associated with AMD development [17,43]; however, the function and implication of these variants in AMD pathogenesis are not fully understood. Age is the strongest environmental risk factor for AMD [3], associated with increased oxidative stress resulting in oxidized proteins and lipids in the aged retina and RPE [44]. Cigarette smoking also intensifies oxidative stress due to its strong oxidant chemical components [2], which decrease tissue ascorbic acid and protein sulfhydryl groups, causing oxidation of DNA, lipids, and proteins [2]. Thus, smoking cessation is recommended for patients who are either at risk for AMD or already have AMD [4]. Other risk factors, such as elevated body mass index (BMI), hypertension, history of cardiovascular disease, and plasma fibrinogen, have a moderate association with AMD, and modifying these factors by improving diet and exercise may help prevent or slow down the progression of AMD [3,4,42].

## 3. Transmembrane Protein 97 (TMEM97): A Gene Encoding Sigma 2 (**σ**_2_) Receptor

The sigma (σ) receptors are a distinct class of transmembrane, non-G-protein-coupled receptors consisting of sigma 1 and sigma 2 (σ_1_ and σ_2_) receptors that were discovered in 1976 and 1990, respectively [45,46]. The σ_1_ receptor is a 25 kDa chaperone protein located in the ER and the mitochondria-associated membranes (MAM) and plays an important role in modulating calcium signaling through the IP3 receptor [46,47,48]. In contrast to the σ_1_ receptor, the σ_2_ receptor was poorly characterized for nearly 20 years. It was initially believed to be progesterone receptor membrane component 1 (PGRMC1) protein [49], but two subsequent studies did not support this identification [50,51]. In 2017, Alon et al. resolved the mystery of the σ_2_ receptor by cloning and characterizing it and providing compelling evidence demonstrating that the σ_2_ receptor is encoded by TMEM97, a transmembrane protein in the ER [19]. This landmark study, later confirmed by others [52,53,54], opened the door for in-depth investigations of the mechanisms underlying the actions of σ_2_R/TMEM97 in cellular processes.

**Figure 1:**
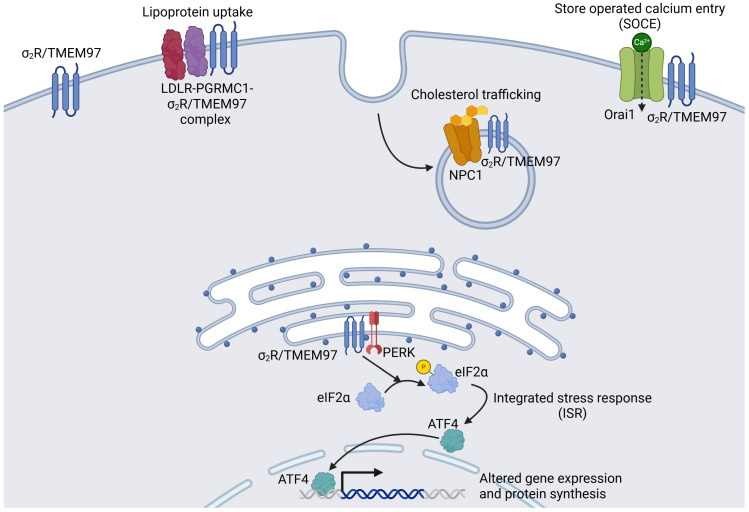
σ_2_R/TMEM97 subcellular localization and function. σ_2_R/TMEM97 is a transmembrane protein primarily found at the endoplasmic reticulum (ER) but can also translocate to the plasma membrane and lysosomes. At the ER, σ_2_R/TMEM97 plays a role in managing cholesterol levels, modulating calcium signaling, and regulating the integrated stress response (ISR) and protein synthesis. At the plasma membrane, σ_2_R/TMEM97 is involved in regulating cholesterol trafficking including the uptake of low-density lipoprotein (LDL) by interacting with progesterone receptor membrane component 1 (PGRMC1) and low-density lipoprotein receptor (LDLR). It also regulates cholesterol transport by interacting with Niemann–Pick C1 (NPC1) at the lysosomes and facilitating the activation of SOCE at the plasma membrane.

σ_2_R/TMEM97 is expressed at high levels in the liver, kidneys, and central nervous system (CNS) [47,55], as well as in cancer cell lines [56,57]. In the eye, σ_2_R/TMEM97 was found in retinal neurons, including retinal ganglion cells (RGCs) and the RPE [19,58]. σ_2_R/TMEM97 is localized subcellularly to the ER as a transmembrane protein, but it can translocate to the plasma membrane and lysosomes [19] (Figure 1). Functionally, σ_2_R/TMEM97 is involved in the regulation of cholesterol homeostasis, autophagy–lysosomal pathway, calcium homeostasis, ISR, and protein synthesis [20,21,22,23]. It plays a crucial role in cholesterol trafficking within the cell, including transport into and out of the ER, to the lysosome, and lipoprotein uptake via the low-density lipoprotein receptor (LDLR); these processes are vital for cell survival and function [19].

## 4. Role of **σ**_2_R/TMEM97 in Cellular Homeostasis

### 4.1. σ_2_R/TMEM97 and Cholesterol Homeostasis

σ_2_R/TMEM97 was identified as a member of the EXPERA (EXPanded EBP superfamily) domain-containing protein family, which consists of proteins including TM6SF1 (Transmembrane 6 Superfamily Member 1), TM6SF2 (Transmembrane 6 Superfamily Member 2), EBP (Emopamil binding protein), and σ_2_R/TMEM97 [59]. The EXPERA domain is believed to possess a sterol isomerase catalytic activity; thus, the proteins containing the EXPERA domain are implicated in sterol metabolism and cholesterol homeostasis [59]. Depletion of cellular sterol activates SREBPs (sterol regulatory element-binding proteins), which are membrane-bound transcription factors that upregulate the expression of genes involved in cholesterol synthesis, including σ_2_R/TMEM97, resulting in increased lipid biosynthesis [60]. Knockout of σ_2_R/TMEM97 decreased cholesterol levels and the rate of internalization of LDL by the LDLR, suggesting that σ_2_R/TMEM97 plays a role in cholesterol homeostasis [60]. Mechanically, σ_2_R/TMEM97 binds to progesterone membrane binding component 1 (PGRMC1) and LDLR and forms a ternary complex to facilitate the rapid internalization of LDL; the formation of this complex, in turn, enhances the LDL uptake [52,61].

In addition to cholesterol synthesis and LDL uptake, σ_2_R/TMEM97 is involved in cholesterol transport out of lysosomes through binding to Niemann–Pick C1 (NPC1). Mutations in the NPC1 gene cause a rare, autosomal recessive, neurodegenerative lysosomal storage disorder, namely Niemann–Pick disease type C1 [21,61]. Knockdown of σ_2_R/TMEM97 upregulates NPC1 expression, reduces cholesterol accumulation, and improves cholesterol trafficking in a cell model of Niemann–Pick type C1 disease [21]. Intriguingly, patients with NPC1 demonstrate signs of retinal neurodegeneration, including a significantly thinner retinal nerve fiber layer (RNFL) and reduced volumes of combined ganglion cell and inner plexiform layer in the macula [62]. Mice lacking the NPC1 gene also develop retinal degeneration, manifested by reduced retinal function, accumulation of lipofuscin in the RPE, and degenerative changes in retinal neurons and their synapses [63]. Future studies are needed to investigate whether enhancing σ_2_R/TMEM97 function can mitigate retinal neuronal injury induced by NPC1 deficiency and improve retinal function.

### 4.2. σ_2_R/TMEM97 and Autophagy

Autophagy is a lysosome-dependent cellular homeostatic process, where damaged or unnecessary cellular components, such as mitochondria, are sequestered by autophagosomes and delivered to lysosomes for degradation and/or recycling [64,65]. Maintaining healthy autophagy is critical for removing toxic waste and recycling essential nutrients to ensure cellular function and survival. Several studies have shown that σ_2_R/TMEM97 plays a critical role in modulating autophagy and influencing lysosomal function [66]. Knockout of σ_2_R/TMEM97 led to impaired autophagy flux and accumulation of autophagosomes, resulting in mitochondrial instability and increased oxidative stress [28,66]. In a model of Parkinson’s disease, a small-molecule modulator of σ_2_R/TMEM97 effectively reduces α-synuclein oligomer toxicity through regulating intracellular lipid vesicle trafficking, autophagy, and cholesterol metabolism [67]. σ_2_R/TMEM97 may also interact with PGRMC1 to regulate autophagy in the brain and retinal cells [52,68,69]. In addition, σ_2_R/TMEM97 binds to NPC1, whose defects can cause failure in autophagy induction, autophagosomal maturation, and fusion with lysosomes [21].

### 4.3. σ_2_R/TMEM97 and Calcium Homeostasis

σ_2_R/TMEM97 has been shown to regulate calcium homeostasis through activation of the store-operated calcium entry (SOCE) [53,70]. Positive allosteric modulators of σ_2_R/TMEM97 increase transient calcium release from intracellular stores and increase the concentration of intracellular calcium [70]. σ_2_R/TMEM97 modulation decreases SOCE and increases apoptosis of cancer cells [53,70]. In MDA-MB-23 breast cancer cells, a triple-negative breast cancer cell line, σ_2_R/TMEM97 overexpression decreases the inhibitory interaction between cholesterol and SOCE calcium channel Orai1 (ORAI calcium release-activated calcium modulator 1), thus enhancing SOCE; conversely, silencing σ_2_R/TMEM97 increases the inhibitory interaction and suppresses SOCE [22]. These effects were abolished in the cells with a mutant of Orai1, suggesting that the action of σ_2_R/TMEM97 in SOCE regulation is dependent on Orai1 [22]. In addition, the novel σ_2_R/TMEM97 fluorescent ligand, NO1, reduces SOCE and impairs the interaction between stromal interaction molecule 1 (STIM1), a protein that senses calcium levels in the ER, and Orai1 [53]. When ER calcium levels drop, STIM1 undergoes conformational changes, clusters, and translocates to the plasma membrane, where it interacts with and activates Orai1, leading to calcium influx into the cell [22,53]. NO1 likely interferes with the positive regulatory effect of σ_2_R/TMEM97 on STIM1, resulting in reduced SOCE [53].

### 4.4. σ_2_R/TMEM97 and Integrated Stress Response (ISR)

The ISR is an adaptive response to cellular stress, such as ER stress, oxidative stress, and accumulation of misfolded proteins, resulting in a decrease in global protein synthesis, while simultaneously initiating the translation of specific mRNAs, such as the mRNA of transcription factor ATF4 (activating transcription factor 4) [71]. While the ISR temporarily reduces protein production and increases activation of certain genes to help restore cellular homeostasis, long-term activation of the ISR and inhibition of protein synthesis can lead to detrimental effects on cellular function and survival and is implicated in a broad range of diseases, such as neurodegenerative disease and cancer [71]. A central step in ISR activation is the phosphorylation of eukaryotic initiation factor 2α (eIF2α) by kinases such as PKR-like ER kinase (PERK), general control nonderepressible 2 (GCN2), heme-regulated eIF2α kinase (HRI), and protein kinase R (PKR) [71]. σ_2_R/TMEM97 modulation with FEM-1689 has been shown to inhibit the ISR, decrease phosphorylated eIF2α levels, and increase neurite outgrowth in mouse dorsal root ganglion neurons [23]. σ_2_R/TMEM97 modulation with FEM-1689 also decreases the ISR and reduces phosphorylated eIF2α levels in human sensory neurons [23]. Conversely, modulation of σ_2_R/TMEM97 with SAS-0132 and DKR-1677 increases the ISR and phosphorylated eIF2α levels in mouse dorsal root ganglion neurons [23,72]. σ_2_R/TMEM97 modulators that decrease ISR and phosphorylated eIF2α levels have antinociceptive properties, whereas modulators that increase ISR and phosphorylated eIF2α levels do not [23]. Collectively, these studies suggest that σ_2_R/TMEM97 is involved in the regulation of ISR with both a positive and negative modulation role; however, the mechanisms underpinning this regulatory action remain largely unknown.

### 4.5. σ_2_R/TMEM97 and Wnt/β-Catenin Signaling

σ_2_R/TMEM97 is overexpressed in various cancers, including epithelial, lung, colorectal, ovarian, and breast cancers, and its overexpression has been linked to poor prognosis and metastasis in some of these cancers [53,73,74,75,76,77]. In breast cancer, σ_2_R/TMEM97 is involved in the activation of the Wnt/β-catenin pathway that regulates cell growth, differentiation, and development [78]. σ_2_R/TMEM97 may interact with the intracellular domain of low-density-lipoprotein-receptor-related protein 6 (LRP6) and enhances LRP6-mediated Wnt signaling in a CK1δ/ε-dependent manner [78]. σ_2_R/TMEM97 knockout in breast cancer cells downregulated the Wnt/β-catenin signaling pathway via LRP6 phosphorylation, suppressing tumor growth [78]. Additionally, silencing σ_2_R/TMEM97 in human gastric cancer cells inhibits cancer cell growth, suggesting that σ_2_R/TMEM97 may be a potential therapeutic target in these types of cancers [79]. Interestingly, σ_2_R/TMEM97 was found to be downregulated in other cancers, such as meningiomas, pancreatic, and renal cancers, suggesting that it may have a complex and cancer-cell-type-dependent role in cancer development [61].

## 5. Role of **σ**_2_R/TMEM97 in Neurodegenerative Diseases

The role of σ_2_R/TMEM97 in neurodegenerative diseases has been explored in the brain and the retina. σ_2_R/TMEM97 expression has been observed in most brain regions, including the hippocampus, dorsomedial hypothalamus, and amygdala, and across all neuronal cell types, including nociceptors, low-threshold mechanoreceptors (LTMRs), and proprioceptors in human and mouse dorsal root ganglia (DRG) and satellite glial cells [23,72,80]. Particularly, σ_2_R/TMEM97 is enriched in proenkephalin (PENK)+ nociceptors and Aδ LTMRs, which are types of neurons involved in pain regulation [23,54,72,81,82]. Global knockout of σ_2_R/TMEM97 shows less anxiety-like and depression-like behaviors in some conditions, such as light/dark preference and tail suspension tests, but not in others, including open field, elevated plus maze, and forced swim tests at baseline [80]. In addition, loss of σ_2_R/TMEM97 reduced long-term neuropathic-pain-induced depression-like phenotype in female mice at 10 weeks after nerve injury [80]. These results suggest that σ_2_R/TMEM97 plays a role in modulating neuropathic-pain-associated anxiety and depression. In primary rat cortical neurons, σ_2_R/TMEM97 was found as a therapeutic target for inhibition of the uptake of neurotoxic peptide Aβ42, whose accumulation and aggregation cause plaque formation and neurodegeneration in Alzheimer’s disease [69]. The study shows that σ_2_R/TMEM97, along with PGRMC1, forms a complex with LDLR, responsible for the uptake and internalization of Aβ42 via apoE-dependent and -independent mechanisms [69]. Thus, targeting this complex may provide a novel approach for preventing and reducing Aβ42-mediated neurotoxicity in Alzheimer’s disease. In the retina, knockout of σ_2_R/TMEM97 does not affect retinal structure and function in normal adult mice but significantly reduces RGC death in ischemic retina [27,29]. These studies support the role of σ_2_R/TMEM97 as a potential therapeutic target in Alzheimer’s disease, neuropathic pain, and ischemic retinopathy.

## 6. Role of **σ**_2_R/TMEM97 in AMD

Recent GWASs and transcriptome-wide association analysis (TWASs) identified σ_2_R/TMEM97 as a putative new AMD risk locus [17,18]. σ_2_R/TMEM97 is expressed in the human retina and RPE [58], and its expression is increased in a mouse model of dry AMD with lipid-rich drusen [83]. In addition to the retina, a critical role of σ2R/TMEM97 in corneal epithelial cell migration has been reported [84]. The exact role of σ_2_R/TMEM97 in RPE pathophysiology is, however, uncertain, and published studies have reported conflicting results [28,30,58]. In one study, σ_2_R/TMEM97 knockout increases the production of reactive oxygen species (ROSs) and decreases the number of photoreceptors in a mouse model of RPE injury [28]. These changes are associated with decreased expression of antioxidant genes nuclear factor–erythroid factor 2-related factor 2 (Nrf2) and superoxide dismutase 2 (SOD2) [28]. In contrast, others demonstrated that knockdown of σ_2_R/TMEM97 by CRISPR interference reduces ROS levels and protects against oxidative-stress-induced cell death in ARPE-19 cells [58]. In a dry AMD model, modulation of σ_2_R/TMEM97 rescued oxidative-stress- and amyloid-beta oligomer (AβO)-induced deficits in the homeostatic recycling of photoreceptor outer segments (POSs) by the RPE [30]; however, how this modulation affects σ_2_R/TMEM97 activity and downstream signaling remains unknown. Nevertheless, these studies support a potential role of σ_2_R/TMEM97 in the regulation of oxidative RPE cell injury. Future research is required to determine the mechanisms underlying the distinct effects of σ_2_R/TMEM97 genetic modification and pharmacological modulation on redox regulation and RPE function in vivo and in vitro.

RPE-derived pro-inflammatory cytokine production can contribute to AMD progression, but the regulation of cytokine production in the RPE is not yet completely understood. A recent study identified a critical role of σ_2_R/TMEM97 in RPE inflammatory factor production through activation of the BAH domain coiled coil 1 (BAHCC1)/NF-κB pathway [31]. σ_2_R/TMEM97 was shown to positively regulate BAHCC1 expression, which is an epigenetic histone reader that increases the expression of transcription factor NF-κB. The activation of NF-κB, in turn, upregulates pro-inflammatory genes, enhancing the production of IL1β and CCL2 [31]. σ_2_R/TMEM97 knockout decreased retinal IL1β and CCL2 expression and alleviated inflammation in a NaIO_3_-induced retinal degeneration model [31]. This study opens a new avenue for studying the role of σ_2_R/TMEM97 as an epigenetic regulator of genes involved in cell pathophysiology, such as oxidative stress and pro-inflammatory cascades, related to AMD and other neurodegenerative diseases.

Drusen formation due to dysregulated cholesterol homeostasis and lipid trafficking is a central step in the development and progression of AMD. A new study revealed that σ_2_R/TMEM97 expression increases during ARPE-19 cell (a human RPE cell line) differentiation [85]. When these cells were treated with a compound that inhibits LDL biosynthesis and lysosomal transport, the LDL level increased, accompanied by enhanced σ_2_R/TMEM97 expression [85]. Furthermore, treatment of cells with σ_2_R/TMEM97 modulators, but not σ_1_R modulators, increased LDL fluorescence, suggesting a role of σ_2_R/TMEM97 in LDL transport in the RPE [85]. Future studies are warranted to determine if modulation of σ_2_R/TMEM97 can regulate LDL trafficking and drusen formation in animal models of AMD in vivo.

Epithelial–mesenchymal transition (EMT) is another important pathological process in the development and progression of AMD [32]. A recent study demonstrated that re-expression of σ_2_R/TMEM97 in the RPE of σ_2_R/TMEM97 knockout mice decreased photoreceptor loss secondary to oxidative damage of the RPE [32]. In ARPE19 cells, knockdown of σ_2_R/TMEM97 activates the cadherin/adhesion-binding pathways, resulting in increased epithelial E-cadherin and mesenchymal N-cadherin expression and partial EMT [32]. Re-expression of σ_2_R/TMEM97 sustains E-cadherin and N-cadherin protein levels through negatively regulating CTNND2 protein [32]. These studies suggest that σ_2_R/TMEM97 may regulate key cellular processes, including oxidative stress, pro-inflammatory cytokine production, cholesterol homeostasis and LDL trafficking, and EMT in RPE, thus playing a crucial role in AMD development and progression.

## 7. **σ**_2_R/TMEM97 Modulators: Potential Treatment for Neurodegenerative Disease and Beyond 

Given the important role of σ_2_R/TMEM97 in cellular processes, small-molecule modulators of σ_2_R/TMEM97 have been generated and tested for their therapeutic potential in human diseases (Table 1). The orientations of the protonated amino groups and the hydrophobic aryl groups determine the binding affinity of σ_2_R/TMEM97 modulators; thus, slight changes within the various modulators can have significant effects on binding [86]. A randomized controlled clinical trial in 2018 investigated the effects of modulating σ_2_R/TMEM97 with MIN-101, which combines a negative allosteric modulator of σ_2_R with a 5-HT2A antagonist, in patients with negative symptoms of schizophrenia [87]. The results demonstrate a statistically significant benefit of MIN-101 over a placebo in improving cognitive performance in these individuals [87]. Animal studies show that the σ_2_R/TMEM97 modulator JVW-1034 decreases withdrawal-related excessive alcohol intake in a *Caenorhabditis elegans* model and a rodent model of alcohol dependence [88]. This effect is specific for alcohol intake and preference without affecting water, total fluid, food, or sucrose intake [89]. JVW-1034 also decreases thermal hyperalgesia and hypersensitivity in alcohol-withdrawn mice [89]. These studies suggest that σ_2_R/TMEM97 modulation may provide a promising approach for treatment of alcohol use disorder.

σ_2_R/TMEM97 modulators have demonstrated neuroprotective effects in various models of Alzheimer’s disease, Parkinson’s disease, and Huntington’s disease. A study found that multiple σ_2_R/TMEM97 modulators with similar structures act as Aβ oligomer antagonists and effectively block the harmful effects of Aβ oligomers [90]. CT1812, a σ_2_R/TMEM97 modulator, can reduce the interaction of Aβ oligomers with σ_2_R/TMEM97, increase the clearance of Aβ oligomers, improve synaptic function, and thus boost cognitive performance in mouse models of Alzheimer’s disease [91]. These findings are supported by a recent human stem cell study that identified a direct relationship between Aβ and σ_2_R/TMEM97 in neuronal synapses in Alzheimer’s brain tissue and showed that CT1812 treatment increases the expression of genes involved in synaptic function [25]. Another study found that the σ_2_R/TMEM97 negative allosteric modulator SAS-0132 is neuroprotective with enhanced cognitive and anti-inflammatory effects in an Alzheimer’s disease and healthy wild-type mouse model [92]. In addition, σ_2_R/TMEM97 modulators have shown beneficial effects in reducing the neurotoxicity caused by α-synuclein oligomers in a Parkinson’s disease model [67] and by human mutant huntingtin (mHTT) protein in a Huntington’s disease model [26].

In blast and controlled cortical impact injury models of traumatic brain injury in mice, it has been shown that σ_2_R/TMEM97 modulation with DKR-1677 decreases axonal degradation, increases cortical neuron survival, and preserves cognition [27]. σ_2_R/TMEM97 modulation with DKR-1677 also decreased RGC degeneration in a mouse model of retinal ischemic injury [29]. Modulation of σ_2_R/TMEM97 by small-molecule ligands has shown analgesic effects in mouse models of neuropathic pain, and this effect requires the presence of the σ_2_R/TMEM97 gene, suggesting that the compounds specifically target σ_2_R/TMEM97 [23,72]. σ_2_R/TMEM97 modulation with FEM-1689 decreases phosphorylated eIF2α levels and inhibits the ISR, whereas σ_2_R/TMEM97 modulation with DKR-1677 increases phosphorylated eIF2α levels and the ISR, illustrating the variable effects of positive and negative allosteric modulators on σ_2_R/TMEM97 [23].

In an illumination-induced mouse model of AMD, modulation of σ_2_R/TMEM97 with CM398 decreased photoreceptor loss and reduced autofluorescent granule formation, a characteristic change of RPE damage [93]. In another study using a human RPE cell model, the σ_2_R/TMEM97 modulator CT1812 was found to rescue both Aβ-mediated and oxidative-stress-induced deficits in photoreceptor outer segment trafficking, restoring it to control levels [85]. CT1812 is currently being investigated in clinical trials for Alzheimer’s disease, Lewy body dementia, and geographic atrophy secondary to dry AMD [85]. The results may thus provide important information on whether σ_2_R/TMEM97 modulation may provide a beneficial effect in a wide range of neurodegenerative diseases, specifically AMD.

Collectively, the results summarized herein provide important information suggesting that σ2R/TMEM97 modulation may provide beneficial effects in a wide range of neurological disorders, including the negative symptoms of schizophrenia, alcohol use disorder, neuropathic pain, and neurodegenerative diseases such as Alzheimer’s, Parkinson’s, and Huntington’s diseases, as well as AMD.

## 8. Conclusions and Future Directions

AMD is the most prevalent cause of vision impairment in the aging population. While significant progress has been made in treating wet AMD, there is still a significant deficit in effective treatments for dry AMD, particularly in its early stages. The newly FDA-approved treatments targeting the complement system can only slow down the progression of GA but do not improve vision. Thus, there is a significant need for effective therapies to manage and treat dry AMD. σ_2_R/TMEM97 was recently identified as a critical regulator of cholesterol homeostasis, lysosome autophagy, calcium homeostasis, and ISR. In addition, multiple lines of evidence from genetic studies, cell studies, animal studies, and clinical trials strongly suggest a role of σ_2_R/TMEM97 in AMD and neurodegenerative diseases of the CNS. Furthermore, there is growing evidence of shared mechanisms between AMD and other neurodegenerative diseases like Alzheimer’s and Parkinson’s disease, including pathophysiological pathways related to inflammation, oxidative stress, and impaired autophagy. The shared pathologies, such as the presence of amyloid-beta in drusen in AMD patients, and common risk factors, including aging and genetic variants in CFH and ApoE4 in AMD and Alzheimer’s disease, suggest potential overlap in disease pathogenesis. Given that σ_2_R/TMEM97 is implicated in the regulation of lipid metabolism, autophagy, oxidative stress, inflammation, and ISR, it is expected that future research exploring σ_2_R/TMEM97 modulation will provide valuable insights into its role in these critical pathways and potentially lead to new therapeutic strategies for AMD. Beyond AMD, perturbation of lipid metabolism, imbalance of autophagy, enhanced oxidative stress and inflammation, as well as dysregulation of the ISR are key factors involved in the pathogenesis of many ocular diseases. These include, but are not limited to, chronic retinal diseases, such as retinitis pigmentosa, diabetic retinopathy, and ischemic retinal disease. Recent work suggests that σ_2_R/TMEM97 is a promising target for developing neuroprotective treatment for ischemic retinal disease. However, the exact roles of σ_2_R/TMEM97 in retinal neurons and neurodegenerative retinal diseases remain to be investigated. In the cornea, σ_2_R/TMEM97 interacts with histain-1, promoting corneal epithelial cell migration. Targeting the σ_2_R/TMEM97 pathway may therefore provide an alternative approach for enhancing corneal wound healing and treating corneal diseases such as diabetic corneal neuropathy.

Although multiple small-molecule compounds that bind to and modulate σ_2_R/TMEM97 function demonstrate beneficial effects on neuroprotection in dry AMD models and age-related neurodegenerative diseases, gain-of-function or loss-of-function studies using cell or animal models with genetic intervention of σ_2_R/TMEM97 show contradictory results on how σ_2_R/TMEM97 regulates oxidative stress and cell survival of the RPE. The inconsistency and complexity of the findings from σ_2_R/TMEM97 modulators and genetic modifications may indicate a cell-type-dependent and condition-dependent role of σ_2_R/TMEM97 in vivo. The contributing factors to these discrepancies may include but are not limited to different stressors used to induce oxidative damage [e.g., sodium iodate vs. tert-butyl hydroperoxide (tBHP)], different cell types (e.g., RPE cells vs. neurons and other cell types) where σ_2_R/TMEM97 may play distinct roles, and different approaches of intervention (e.g., genetic knockout models vs. pharmacological modulators). For the latter, global knockout mice σ_2_R/TMEM97 were used in most reported studies in the RPE and retina. The close interactions between the RPE and retinal neurons and the feedback regulations of signaling pathways involved in maintaining the RPE and retinal metabolic and functional homeostasis may generate a substantial impact on the study outcome. In addition, the σ_2_R/TMEM97 ligands with even subtle differences in their structures exhibit diverse effects on the modulation of neuronal survival and function, and the molecular mechanisms underlying these differences are not fully understood. Future studies are warranted to elucidate the fundamental principle in the context of the structure–function relationship of σ_2_R/TMEM97 modulators in regulating σ_2_R/TMEM97 activity and downstream signaling pathways. This information will provide potential insights into the diverse role of σ_2_R/TMEM97 in different cell types and diseases, facilitating the development of new drug treatments in AMD and neurodegenerative diseases. Furthermore, σ_2_R/TMEM97 is involved in a wide range of cellular processes. As such, developing new bioactive σ_2_R/TMEM97 modulators with higher selectivity for σ_2_R/TMEM97 may reduce the potential off-target effects, improving the potential of using non-invasive delivery routes such as systemic or topical administration for the treatment of AMD. In addition, with future research to better understand the complex, and potentially cell-type-specific, roles of σ_2_R/TMEM97 in the RPE and retina, using genetic approaches targeting σ_2_R/TMEM97, such as adeno-associated virus (AAV)-mediated gene delivery or gene editing technologies, may provide alternative strategies for developing new treatments for retinal diseases.

## Figures and Tables

**Table 1:** Summary of the current research on σ_2_R/TMEM97 modulators.

Pathology	Model	Pathway	Effect on Pathway	Resources
Schizophrenia	σ_2_R/TMEM97 modulator MIN-101 clinical trail	Cognitive performance	Increases	[87]
Alcohol dependence	σ_2_R/TMEM97 modulator JVW-1034 in a rodent model	Alcohol-withdrawal-induced excessive alcohol consumption, alcohol intake, and associated pain states	Decreases	[88,89]
Alzheimer’s disease	σ_2_R/TMEM97 modulator CT01344 in cell and mouse models	Amyloid beta (Aβ) accumulation and synaptotoxicity	Decreases	[90]
Alzheimer’s disease	σ_2_R/TMEM97 modulator CT1812 in preclinical cell and mouse models, clinical trial, and human stem cells	Amyloid beta (Aβ) accumulation and synaptotoxicity	Decreases	[25,91]
Alzheimer’s disease	σ_2_R/TMEM97 modulator SAS-0132 in a transgenic mouse model of Alzheimer’s disease	Neuroprotection, cognitive performance, and inflammation	Decreases	[92]
Parkinson’s disease	σ_2_R/TMEM97 modulation in rat neuron and glial cell culture	α-synuclein accumulation and neurotoxicity	Decreases	[67]
Huntington’s disease	σ_2_R/TMEM97 modulation in a neuron cell model	Neuronal toxicity	Decreases	[26]
Traumatic brain injury	σ_2_R/TMEM97 modulator DKR-1677 in mouse models of traumatic brain injury	Neuronal degeneration	Decreases	[27]
Ischemic retinal ganglion cell (RGC) injury	σ_2_R/TMEM97 modulator DKR-1677 in an ischemia-induced RGC degeneration mouse model	Ischemic injury causing RGC degeneration	Decreases	[29]
Neuropathic pain	σ_2_R/TMEM97 modulators FEM-1689, UKH-1114, Z1665845742, and Z4857158944 in a spared nerve injury mouse model; knockout in neuron cell and mouse models	Activation of integrated stress response (ISR) drives neuropathic pain	Decreases	[23,54,72]
Neuropathic pain	σ_2_R/TMEM97 modulator CM398 in mouse model	Formalin and thermal models; chronic nerve constriction injury	Decreases	[81,82]
AMD	σ_2_R/TMEM97 modulator CM398 in mouse model	Photoreceptor loss	Decreases	[93]
AMD	σ_2_R/TMEM97 modulator CT1812 in a mouse model and a human RPE model	Aβ-mediated and oxidative stress-mediated photoreceptor outer segment (POS) trafficking	Decreases	[85]

## Data Availability

No new data were created or analyzed in this study.
